# Differences in beliefs and home environments regarding energy balance behaviors according to parental education and ethnicity among schoolchildren in Europe: the ENERGY cross sectional study

**DOI:** 10.1186/1471-2458-14-610

**Published:** 2014-06-17

**Authors:** Johannes Brug, Léonie Uijtdewilligen, Maartje M van Stralen, Amika S Singh, Mai JM ChinAPaw, Ilse De Bourdeaudhuij, Nanna Lien, Elling Bere, Lea Maes, Juan M Fernández-Alvira, Nataša Jan, Eva Kovacs, Alain Dössegger, Yannis Manios, Saskia J te Velde

**Affiliations:** 1 Department of Epidemiology and Biostatistics and EMGO Institute for Health and Care Research, VU University Medical Center, P.O. Van der Boechorststraat 7, Amsterdam, BT 1081, the Netherlands; 2 Department of Public and Occupational Health and EMGO Institute for Health and Care Research, VU University Medical Center, Amsterdam, the Netherlands; 3 Department of Movement and Sport Sciences, Ghent University, Ghent, Belgium; 4 Department of Nutrition, University of Oslo, Oslo, Norway; 5 Department of Public Health, Sport and Nutrition, University of Agder, Kristiansand, Norway; 6 Department of Public Health, Ghent University, Ghent, Belgium; 7 GENUD (Growth, Exercise, Nutrition and Development) Research Group. E.U. Ciencias de la Salud, Universidad de Zaragoza, Zaragoza 50009, Spain; 8 Slovenian Heart Foundation, Ljubljana, Slovenia; 9 Department of Paediatrics, Pecs University, Pecs, Hungary; 10 Swiss Federal Institute of Sport Magglingen SFISM, Ittigen, Switzerland; 11 Department of Nutrition and Dietetics, Harokopio University, Athens, Greece

**Keywords:** Schoolchildren, Home environments, Beliefs, Parental education, Ethnicity, Energy-balance behaviors

## Abstract

**Background:**

To explore differences in personal and home environmental factors that are regarded as determinants of energy balance-related behaviors (EBRBs) according to parental education and ethnic background among 10–12 year old schoolchildren across Europe.

**Methods:**

A school-based survey among 10–12 year olds was conducted in eight countries across Europe. A range of personal and home environment variables relevant for soft drink consumption, daily breakfast, sport participation and TV time was assessed by means of child report. Personal factors included attitude, health beliefs, and preference/liking. Home environment factors included parental subjective norm, modeling, support, practices and home availability. Children were classified based on parental education (i.e., low vs. high) and ethnic background (i.e., native vs. non-native). Data from 6018 children originating from 83 schools were included in the analyses.

**Results:**

Multilevel logistic regression analyses showed that the majority of the factors tested –and especially home environment variables- were more favorable among children from higher educated parents and from native ethnicity. None of the personal and home environment factors was found to be more favorable among children from lower educated parents or non-native ethnicity.

**Conclusions:**

The present study indicates that schoolchildren from lower educated and non-native parents across Europe have EBRB-related beliefs and are exposed to home environments that are less favorable for engagement in healthy EBRBs.

## Background

Recent research shows that prevalence of overweight and obesity, as well as prevalence of behavioral risk factors for overweight and obesity among school-aged children are high across Europe, but also differ considerably between European countries [1]. Reviews and original research suggest that intakes of sugar sweetened beverages, breakfast skipping, physical activity, and sedentary behavior are important energy balance-related behaviors (EBRBs) among school-aged children [2-4]. The results of the cross-sectional study that is part of the ENERGY-project [1,5,6], showed large differences in prevalence of overweight and obesity (44.4% among boys in Greece to 13.5% among girls in Belgian Flanders) and EBRBs between countries -with in general more favorable patterns in northern European countries-, as well as differences according to parental education and ethnicity across these countries [1,5]. Other studies have also found large differences in childhood overweight and obesity according to parental education and ethnicity in countries in Europe and beyond [7-24]. Preventing overweight and obesity and promoting healthy EBRBs in youth is thus important for promotion of population health, and should especially be aimed at vulnerable groups, such as lower socio-economic status groups and ethnic minorities.

To tailor health promotion and obesity prevention interventions to the most vulnerable groups, insight in differences in potential behavioral determinants, e.g., in the children’s EBRB-specific motivations, abilities and perceived opportunities according to level of education and ethnicity, is needed [25,26]. For school-aged children, personal motivational factors and school environments have been studied extensively and identified as important determinants of EBRBs [27-29]. Home environmental factors have been studied less but are also of particular importance [30,31] and are a main focus of the present investigation.

The current study explores differences in personal and home environment factors that are regarded as potential determinants of EBRBs in schoolchildren according to parental education and ethnicity across Europe. The specific research question is: ‘What are the differences in personal and home environment factors that are regarded as potential determinants of physical activity, sedentary and dietary behaviors according to parental education and ethnic background among 10–12 year old schoolchildren across eight countries in Europe?’

## Methods

A description of the rationale and organization of the ENERGY-project [31] and a comprehensive description of the design, procedures, and methodology of the ENERGY school-based survey are published elsewhere [6]. The data collection protocol and survey questionnaires for the ENERGY cross-sectional survey are available online at http://www.projectenergy.eu (in English and the 7 languages in which the questionnaire was administered). The studies were approved by the corresponding local ethics committees in all participating countries. In Belgium the survey was approved by the Medical Ethics Committee of the University Hospital Ghent; In Greece the survey was approved by the Bioethics Committee of Harokopio University; In Hungary the survey was approved by the Scientific and Ethics Committee of Health Sciences Council; In the Netherlands the survey was approved by the Medical Ethics Committee of the VU University Medical Center; In Norway the survey was approved by the National Committees for Research Ethics in Norway; In Slovenia the survey was approved by the National Medical Ethics Committee of the Republic of Slovenia; In Spain the survey was approved by the Clinical Research Ethics Committee of the Government of Aragon; In Switzerland study was approved by the ethics committees of the participating cantons (Basel, Bern, Aargau and St. Gallen).

### Sampling and respondents

Between March and July 2010 the ENERGY school-based survey was carried out in Belgium, Greece, Hungary, the Netherlands, Norway, Slovenia and Spain. Data collection in Switzerland was conducted between June and December 2010. Across the countries, 1000 pupils aged 10–12 and one parent/caregiver for each child per country was aimed for. In Greece, Hungary, the Netherlands, and Slovenia, sampling was done nationally, while in Spain, Belgium, Norway and Switzerland schools were selected in the region of Aragón, Flanders, the southern regions, and German speaking regions, respectively. More details regarding recruitment are described in open access journals elsewhere [6,32]. Response rates at the child level were very high (82-100%) for children for whom parental consent was obtained, but mainly because of parents not returning completed parental consent forms, the net response rate was 50% or lower in Hungary (33%), Norway (45%), Spain (43%) and Switzerland (50%). For the parent questionnaire response rates ranged between 40% in the Netherlands and Spain, and 86% in Slovenia [1].

### Measures

Detailed information regarding the procedures, training of research staff, the development of questionnaires [6], and test-retest reliability and construct validity of the questionnaires are published in open access journals elsewhere [33,34]; good to excellent intra-class coefficients were found for the vast majority of questionnaire items. The schoolchildren completed questionnaires and anthropometric measurements during school time. Parents received and returned the parent questionnaire via their children. Measurements were conducted according to a standardized protocol.

#### Personal and home environment variables

For the present study we examined several personal and home factors that are regarded as correlates and determinants of soft drinks consumption, eating breakfast, engaging in sports and screen viewing (TV and PC time) and that were assessed in the child questionnaire. All these constructs, their description and the questionnaire items have been reported in detail previously [6].

Personal factors included attitude, health beliefs, and preference/liking. Home environment factors included parental subjective norm, parent modeling, co-participation in the EBRB, active encouragement/parental support, parenting practices, and home availability. The relevant questionnaire items are presented in Additional file 1. Attitude, health beliefs, parenting practices, and home availability were assessed for each behavior separately. For soft drinks consumption children did not provide data regarding co-participation in the EBRB and active encouragement/parental support. For screen viewing behavior children were not asked about active encouragement/parental support. All variables were dichotomized into positive or favorable vs. negative or unfavorable values (i.e., in the sense of supportive or unsupportive for healthy behavior; Additional file 1) because of skewed distributions of most of the variables.

#### Parental education and ethnic background

Parents were asked to report their own level of education and that of the other parent/caregiver in the parent questionnaire. Answer categories were less than 7 years, 7–9 years, 10–11 years 12–13 years and 14 years or more. Parental education was dichotomized into low (both parents/caregivers with fewer than 14 years of education) and high (at least one parent/caregiver with 14 or more years of education), which approximately distinguishes families with at least one caregiver who completed medium or higher vocational, college or university training from other families. We used years of education because this question could be asked in all participating countries where different school systems and degree levels are in place.

Ethnic background was assessed in two ways. First, children reported the language mostly spoken at home, with the answering options tailored to the different countries (i.e., answering options including the official language or languages of the specific country or region, the native languages of the largest ethnic minorities, and a category ‘other’). Children who reported to primarily speak the official language of the country of administration (e.g., Greek in Greece; Dutch in the Netherlands) were classified as ‘native’. All other children were classified as ‘non-native’. Second, parents indicated whether the biological parents of their child were born in the country of administration. Answering options were yes; no, only one parent and; no, none of the parents. A dichotomous variable was created to distinguish parents with a ‘native’ background (i.e., both parents were born in the country of administration) from those with a ‘non-native’ background (i.e., at least one parent was born in another country). Country of birth of parents has been used to assess ethnic background or immigrant status in other recent cross-European research [35].

### Statistics

Descriptive statistics were performed using IBM SPSS Statistics version 20.0. Differences in personal and home variables regarding soft drinks consumption, breakfast, sports participation and screen viewing behavior according to parental education and ethnic background were tested by performing multilevel logistic regression (levels: country, school, individual) with a random intercept for country and school and using a second order Penalized Quasi-Likelihood (PQL) estimation procedure in MLwiN (version 2.18). Both age and gender were included as covariates, and all analyses on ethnic differences were conducted with and without adjustment for parental education.

Subjects were excluded from analyses if they did not provide data on any of the correlates presently examined, or if they could not be matched with parental data on education or ethnic background. Chi-square tests were performed to check for significant differences (i.e., bias) between included and excluded subjects.

Parameter estimates of the regression analyses were expressed as Odds Ratios (OR) with a 95% confidence interval (95% CI), and results were considered significant if *p* < 0.05.

## Results

### Participant characteristics

Of the 7915 children (52% girls; mean age = 11.7 ± 0.8 years) who completed the ENERGY questionnaire, 99 did not provide any data on correlates that were included in the present study. Data of another 1798 children could not be matched with data on either parental education or ethnic background. Hence, the total sample for the present study comprised 6018 children (53% girls, mean age = 11.6 ± 0.7 years). Of those, 6000 provided data on language mostly spoken at home, and 5977 had available data on country of birth of parents. Thirty-nine percent of the total sample had two parents/caregivers with fewer than 14 years of education. Regarding ethnicity, 6% reported *not* to primarily speak the official language of the country of administration, and 17% were classified as non-native based on their parents country of birth. Additional file 2 displays the distribution of parental education and ethnic background by country.

In general, excluded subjects were less likely to report favorable personal and home environment variables. Parents of older children, boys and non-natives based on language spoken at home were less likely to complete the parent questionnaire.

### Differences according to parental education

Children from higher educated parents were in general more likely to report favorable personal and home environment variable values across all EBRBs than children from lower educated parents (Table 1). Of the 42 variables examined, 26 showed results in this direction. This pattern was particularly evident with regard to TV viewing and sport participation. The remaining 16 variables showed no significant differences.

**Table 1:** Multilevel binary logistic regression analyses testing for differences according to parental education

**N = 6018**		
**Correlate**	**OR**^**a**^	**95%CI**
**Soft drink**		
Unfavorable attitude	0.64***	**(0.55; 0.75)**
Incorrect health beliefs	0.94	(0.81; 1.09)
High preference/liking	0.89	(0.77; 1.02)
Unfavorable parental subjective norm	0.72***	**(0.59; 0.87)**
Low parent modeling	0.75***	**(0.64; 0.87)**
Parental practices		
*Rules*	0.86*	**(0.76; 0.97)**
*High allowance*	0.75***	**(0.65; 0.86)**
*Bought on request*	0.95	(0.83; 1.08)
*High accessibility*	0.80**	**(0.70; 0.91)**
High home availability	0.73***	**(0.65; 0.83)**
**Breakfast**		
Unfavorable attitude^b^	0.77	(0.41; 1.47)
Health beliefs		
*Incorrect (eating breakfast)*	0.71***	**(0.59; 0.86)**
*Incorrect (not eating breakfast)*	1.08	(0.96; 1.23)
Low preferences/liking	0.96	(0.73; 1.25)
Unfavorable parental subjective norm^b^	0.70	(0.31; 1.57)
Low parent modeling	0.85	(0.68; 1.07)
Low co-participation	0.77***	**(0.68; 0.88)**
Low levels of active encouragement/parental support	0.93	(0.79; 1.09)
Parental practices		
*Rules*	0.84**	**(0.74; 0.94)**
*Bought on request*	0.86**	**(0.76; 0.96)**
Low home availability^b^	1.03	(0.65; 1.65)
**Physical activity/sports**		
Unfavorable attitude^b^	0.37	(0.08; 1.70)
Incorrect health beliefs	0.72***	**(0.61; 0.85)**
Low preferences/liking^b^	0.73	(0.42; 1.26)
Unfavorable parental subjective norm^b^	0.75	(0.35; 1.61)
Low parent modeling	0.69***	**(0.60; 0.80)**
Low co-participation	0.85*	**(0.74; 0.96)**
Low levels of active encouragement/parental support	0.95	(0.76; 1.20)
Parental practices		
*Rules*	0.86*	**(0.75; 0.97)**
*Low general allowance*^b^	0.60*	**(0.41; 0.89)**
*Low specific allowance*^b^	0.70*	**(0.49; 1.00)**
Low home availability	0.64***	**(0.56; 0.74)**
**TV Viewing**		
Unfavorable attitude	0.85	(0.72; 1.00)
Incorrect health beliefs	0.69***	**(0.61; 0.78)**
High preferences/liking	1.06	(0.93; 1.21)
Unfavorable parental subjective norm	0.65***	**(0.52; 0.81)**
High parent modeling	0.75***	**(0.67; 0.85)**
High co-participation	0.66***	**(0.56; 0.76)**
Parental practices		
*Rules*	0.76***	**(0.67; 0.85)**
*High general allowance*	0.63***	**(0.56; 0.72)**
*High specific allowance*	0.70***	**(0.62; 0.79)**
High home availability	0.41***	**(0.36; 0.47)**

^a^Odds ratios (OR) and 95% confidence intervals (CI) as derived from multilevel binary logistic regression analyses, adjusted for age and gender, testing for differences in likelihood to report favorable or unfavorable personal and home variables regarding soft drink consumption, breakfast, participation in sports, and TV time among 10–12 year old children from lower educated parents compared to children from higher educated parents. An OR >1 indicates that children with high educated parents are more likely to be in the correlate category coded 1 (see Additional file 1 for categorization); an OR <1 indicates that children with high educated parents are less likely to be in the correlate category coded 1 (see Additional file 1 for categorization). Statistical significant ORs are printed in bold, *p < 0.05, **p < 0.01, ***p < 0.001.

^b^Due to small numbers in one of the two categories, multilevel analysis could not be performed using a second order Penalized Quasi-Likelihood (PQL) estimation procedure. Instead a first order Maximum Quasi-Likelihood (MQL) estimation procedure was used for these potential correlates.

### Differences according to ethnic background

Native children -based on language spoken at home as well as based on country of birth of the parents- were in general more likely to report favorable personal and home environment variable values across all EBRBs than non-native children (Table 2). Of the 42 variables examined 16 and 13 –for language spoken at home an country of birth of parents, respectively- showed such results; the other variables were not significantly different between native and non-native schoolchildren. Again, the pattern was particularly evident with regard to TV viewing. When adjusted for parental education, the majority of the differences remained statistically significant.

**Table 2:** Multilevel binary logistic regression analyses testing for differences according to ethnic background

	**Language spoken at home (N = 6000)**				**Country of birth of biological parents (N = 5977)**			
	**Unadjusted**		**Adjusted**		**Unadjusted**		**Adjusted**	
**Correlate**	**OR**^**a**^	**95%CI**	**OR**^**a**^	**95%CI**	**OR**^**a**^	**95%CI**	**OR**^**a**^	**95%CI**
**Soft drink**								
Unfavorable attitude	0.73*	**(0.56; 0.96)**	0.80	(0.61; 1.05)	0.69***	**(0.57; 0.84)**	0.73***	**(0.60; 0.88)**
Incorrect health beliefs	1.19	(0.89; 1.59)	1.21	(0.90; 1.61)	1.11	(0.92; 1.35)	1.12	(0.92; 1.36)
High preference/liking	1.02	(0.80; 1.31)	1.03	(0.80; 1.33)	1.04	(0.88; 1.23)	1.06	(0.90; 1.26)
Unfavorable parental subjective norm	0.80	(0.56; 1.15)	0.86	(0.60; 1.24)	0.82	(0.64; 1.05)	0.86	(0.67; 1.10)
Low parent modeling	1.10	(0.79; 1.52)	1.16	(0.84; 1.61)	0.89	(0.72; 1.09)	0.92	(0.75; 1.13)
Parental practices								
*Rules*	0.98	(0.78; 1.22)	1.01	(0.80; 1.26)	1.14	(0.98; 1.32)	1.16*	**(1.00; 1.35)**
*High allowance*	0.80	(0.62; 1.02)	0.84	(0.66; 1.07)	0.98	(0.82; 1.17)	1.02	(0.85; 1.21)
*Bought on request*	0.83	(0.65; 1.05)	0.84	(0.66; 1.07)	0.89	(0.76; 1.05)	0.90	(0.76; 1.06)
*High accessibility*	1.04	(0.80; 1.34)	1.09	(0.84; 1.41)	1.17	(0.98; 1.39)	1.21*	**(1.01; 1.45)**
High home availability	0.99	(0.78; 1.25)	1.05	(0.83; 1.32)	1.06	(0.91; 1.24)	1.11	(0.95; 1.29)
**Breakfast**								
Unfavorable attitude^b^	1.58	(0.35; 7.19)	1.68	(0.37; 7.73)	1.00	(0.44; 2.27)	1.04	(0.45; 2.39)
Health beliefs								
*Incorrect (eating breakfast)*	0.62**	**(0.44; 0.86)**	0.66*	**(0.47; 0.92)**	0.75*	**(0.59; 0.96)**	0.79	(0.62; 1.00)
*Incorrect (not eating breakfast)*	1.21	(0.96; 1.52)	1.19	(0.95; 1.50)	1.03	(0.88; 1.20)	1.02	(0.87; 1.19)
Low preferences/liking	0.84	(0.52; 1.36)	0.85	(0.52; 1.38)	1.06	(0.75; 1.49)	1.07	(0.75; 1.51)
Unfavorable parental subjective norm^b^	0.61	(0.17; 2.26)	0.66	(0.18; 2.47)	0.51	(0.21; 1.23)	0.53	(0.21; 1.30)
Low parent modeling	1.13	(0.74; 1.73)	1.17	(0.76; 1.80)	1.27	(0.95; 1.70)	1.31	(0.97; 1.76)
Low co-participation	0.94	(0.74; 1.20)	0.99	(0.78; 1.27)	0.92	(0.79; 1.08)	0.96	(0.81; 1.12)
Low levels of active encouragement/parental support	0.99	(0.74; 1.34)	1.01	(0.75; 1.36)	0.86	(0.70; 1.04)	0.87	(0.71; 1.05)
Parental practices								
*Rules*	0.76*	**(0.60; 0.97)**	0.79*	**(0.62; 1.00)**	0.86	(0.74; 1.00)	0.88	(0.76; 1.03)
*Bought on request*	0.66***	**(0.53; 0.82)**	0.67***	**(0.54; 0.84)**	0.87	(0.75; 1.01)	0.89	(0.77; 1.03)
Low home availability^b^	0.51	(0.26; 1.01)	0.50*	**(0.25; 1.00)**	0.69	(0.41; 1.17)	0.68	(0.40; 1.17)
**Physical activity/sports**								
Unfavorable attitude^c^	-	-	-	-	-	-	-	-
Incorrect health beliefs	0.56***	**(0.43; 0.74)**	0.60***	**(0.46; 0.79)**	0.74**	**(0.61; 0.90)**	0.78*	**(0.64; 0.95)**
Low preferences/liking^b^	0.46	(0.20; 1.02)	0.49	(0.22; 1.11)	0.77	(0.40; 1.49)	0.81	(0.42; 1.57)
Unfavorable parental subjective norm^b^	2.17	(0.27; 17.47)	2.36	(0.29; 19.28)	1.28	(0.45; 3.70)	1.35	(0.46; 3.92)
Low parent modeling	0.70*	**(0.54; 0.90)**	0.74*	**(0.57; 0.96)**	0.73***	**(0.61; 0.86)**	0.76**	**(0.64; 0.91)**
Low co-participation	0.98	(0.76; 1.26)	1.02	(0.79; 1.31)	1.02	(0.86; 1.20)	1.04	(0.95; 1.14)
Low levels of active encouragement/parental support	0.78	(0.53; 1.14)	0.79	(0.54; 1.15)	0.80	(0.61; 1.04)	0.80	(0.61; 1.05)
Parental practices								
*Rules*	0.93	(0.73; 1.18)	0.96	(0.75; 1.22)	0.97	(0.83; 1.13)	0.99	(0.85; 1.15)
*Low general allowance*^b^	0.48*	**(0.27; 0.88)**	0.55*	**(0.30; 1.00)**	0.62*	**(0.39; 0.97)**	0.67	(0.43; 1.06)
*Low specific allowance*^b^	0.60	(0.34; 1.06)	0.65	(0.36; 1.15)	0.77	(0.50; 1.18)	0.82	(0.53; 1.26)
Low home availability	0.43***	**(0.33; 0.56)**	0.46*	**(0.36; 0.60)**	0.52***	**(0.43; 0.62)**	0.54***	**(0.45; 0.64)**
**TV Viewing**								
Unfavorable attitude	0.43***	**(0.32; 0.58)**	0.44***	**(0.33; 0.59)**	0.72**	**(0.58; 0.89)**	0.73**	**(0.59; 0.91)**
Incorrect health beliefs	0.57***	**(0.46; 0.72)**	0.61***	**(0.49; 0.77)**	0.60***	**(0.51; 0.70)**	0.62***	**(0.53; 0.73)**
High preferences/liking	0.93	(0.72; 1.18)	0.91	(0.71; 1.17)	0.88	(0.74; 1.04)	0.87	(0.74; 1.03)
Unfavorable parental subjective norm	0.51***	**(0.35; 0.74)**	0.55**	**(0.38; 0.81)**	0.72*	**(0.55; 0.96)**	0.77	(0.58; 1.02)
High parent modeling	0.59***	**(0.47; 0.73)**	0.61***	**(0.49; 0.77)**	0.81**	**(0.70; 0.94)**	0.84*	**(0.72; 0.97)**
High co-participation	0.68**	**(0.51; 0.91)**	0.73*	**(0.55; 0.98)**	0.96	(0.80; 1.15)	1.01	(0.85; 1.22)
Parental practices								
*Rules*	0.80	(0.64; 1.00)	0.85	(0.68; 1.05)	0.93	(0.80; 1.08)	0.96	(0.83; 1.12)
*High general allowance*	0.64***	**(0.50; 0.81)**	0.69**	**(0.54; 0.88)**	0.74***	**(0.63; 0.87)**	0.78**	**(0.66; 0.92)**
*High specific allowance*	0.67***	**(0.53; 0.84)**	0.71**	**(0.57; 0.90)**	0.72***	**(0.62; 0.84)**	0.76***	**(0.65; 0.88)**
High home availability	0.61***	**(0.48; 0.78)**	0.71**	**(0.56; 0.91)**	0.66***	**(0.56; 0.77)**	0.73***	**(0.62; 0.86)**

^a^Odds ratios (OR) and 95% confidence intervals (CI) as derived from multilevel binary logistic regression analyses, adjusted for age and gender, testing for differences in likelihood to report favorable or unfavorable personal and home variables regarding soft drink consumption, breakfast, participation in sports, and TV time among 10–12 year old children from native compared to non-native origin according to language spoken at home (uncorrected and corrected for parental education). An OR >1 indicates that native children are more likely to be in the correlate category coded 1 (see Additional file 1 for categorization); an OR <1 indicates that native children are less likely to be in the correlate category coded 1 (see Additional file 1 for categorization). Statistical significant ORs are printed in bold, *p < 0.05, **p < 0.01, ***p < 0.001.

^b^Due to small numbers in one of the two categories, multilevel analysis could not be performed using a second order Penalized Quasi-Likelihood (PQL) estimation procedure. Instead a first order Maximum Quasi-Likelihood (MQL) estimation procedure was used for these potential correlates.

^c^Due to small numbers in the category bad – very bad (<1%), multilevel analysis could not be performed.

## Discussion

The present study shows that 10–12 year old children from lower educated parents or those from foreign ethnicity across Europe report less favorable beliefs and especially home environment factors regarding different EBRBs than their peers from higher educated parents and from native ethnicity. It has already been established that children from such vulnerable groups are also more likely to be overweight and obese and to engage in less healthy EBRBs [1,5]. Also based on the ENERGY study data two previous publications have appeared that showed that energy balance behaviors are less favorable among school children from lower educated parents and from ethnic minority groups as compared to those from higher educated and ethnic majority respectively [1,5]. These studies confirmed these differences in this age group, and showed that such differences were very consistent in direction but different in magnitude between different countries in Europe. Because educational and ethnic disparities in health, risk factors and risk behaviors are such an important public health issue, in further studies of the ENERGY data we have tried to explain these differences somewhat further with. In a paper by Jimenez-Pavon et al., we sought to examine the independent associations of parental education and physical activity with children’s physical activity across Europe [36]. In a paper by Alvira-Fernandez et al. in the International Journal of Behavioral Nutrition and Physical Activity [37] we have explored if energy balance behaviors show specific clusters in their association with parental education, and we have tried to explore if these differences in energy balance behaviors mediate differences in body composition in this age group [38].

The present study does not focus on differences in anthropometry or EBRBs, but on such differences in what major health behavior theories and models presume are the individual and environmental level determinants of these behaviors. If such differences are also found in these more upstream factors –and that is indeed what we find in this study- such insights provide entry points for interventions to reduce disparities.

The present study indicates that these children’s home environments are less supportive for healthy EBRBs, and this further suggest that changing such behaviors and reducing the burden of overweight and obesity among these vulnerable groups to contribute to reducing inequalities in these important health determinants, requires involving and inducing changes in the home environment, and such interventions need to be specifically tailored to these socio-demographic groups’ beliefs and environments. However, a recent review shows that the evidence for parental involvement in school-based diet and physical activity health promotion is not consistent, mainly because of lack of well-conducted studies [39]. Further participatory studies among parents and children form these vulnerable groups to explore how parental involvement in such interventions and changes in home environments can be achieved, need to be conducted [40]. Across the behaviors most significant differences were observed in parenting practice, especially for breakfast, sports and TV viewing, and changes in such parenting practices may thus be of specific importance. Interventions focusing on parenting are indeed being conducted to contribute to obesity prevention and treatment [41]. For TV viewing, the majority of concepts explored were significantly different according to ethnicity. Ethnic minority children thus not only have less favorable behavior [5] but also less favorable attitudes, health beliefs, role models, and parenting practices, and interventions need to include personal as well as home environmental changes.

Similar to the differences in overweight prevalence and engagement in EBRBs according to ethnicity previously reported [5], the differences in personal and home environmental factors presented here were in the same direction and of similar magnitude for two different dichotomous indicators of ethnic origin, i.e., language spoken at home and country of birth of the parents. As we have indicated before [5], both indicators are non-specific indicators of ethnic origin –they do not distinguish between the rich diversity in ethnic origins within and across countries in Europe. Despite this obvious limitation, the differences according to ethnicity were apparent and consistent in the different countries included in the present study as well as for a range of personal and home environmental factors, also after adjustment for parental education. This indicates that the ethnic differences are real and robust. Similar to the differences found in overweight and EBRBs [5], the fact that these differences were present even though the ethnic minority group consisted of a range of ethnicities indicates that these differences cannot be explained by ethnic group-specific socio-cultural habits or beliefs. Ethnic minorities in Europe are, for example, on average lower educated, have lower income levels, and more often live in deprived neighborhoods [24]. The results provide further support that this generic vulnerability of people from foreign ethnicity may be the main driver for the differences found; the fact that the differences remained after adjustment for parental education indicates that other socio-economic or social-cultural factors –such as different neighborhood environments, differences in social norms, support or parenting and parental and peer modeling [42-45] - are of importance [46].

In interpreting the results a number of limitations should be considered. Response rates were lower in some countries. This was most probably caused by the fact that these countries required active parental consent. This might have resulted in participation of children whose parents were more interested in issues regarding obesity prevention. Analyses of non-response and missing data analysis showed that parents of older children, boys and non-natives based on language spoken at home were less likely to complete the parent questionnaire. This might have biased the results especially in the countries with low parental response rates. The representation of schoolchildren from non-native ethnicity was comparable to national representative samples [5], but children from lower educated parents were under-represented in the present study [1]. Finally, the personal and home environmental factors studied were all assessed with single item self-reports, and liable for social desirability bias. The measures did in general have good test-retest reliability and construct validity [33,34].

Strengths of the present study include the large multinational sample from different regions across Europe, the standardized data collection protocol across the different countries and the inclusion of a range of personal and home environmental factors.

## Conclusion

The results indicate that schoolchildren across Europe from lower educated and non-native parents have personal beliefs and are exposed to home environments that are not supportive for healthy EBRBs. This indicates that changing such behaviors and reducing the burden of overweight and obesity among these vulnerable groups needs specifically tailored interventions.

## Abbreviations

EBRB: Energy balance-related behaviors.

## Competing interests

The authors declare that they have no competing interests.

## Authors' contributions

JB, SJtV, ASS, MC, IDB, NL, LM, EB, and YM designed the international study. JB, LU, MMVS, IDB, JMF, NL, EB, YM, LM, NJ, EK, AD, SJtV contributed to the development of measurement protocols and instruments and coordinated and supervised the data collections and data management in the participating countries. LU conducted the analyses, supervised by JB and SJtV. JB and LU drafted the manuscript. MMVS, IDB, JMF, NL, EB, YM, LM, NJ, EK, AD, SJtV provided input for the manuscript and approved the submitted manuscript. All authors read and approved the final manuscript.

## Pre-publication history

The pre-publication history for this paper can be accessed here:

http://www.biomedcentral.com/1471-2458/14/610/prepub

## Supplementary Material

Additional file 1 Overview of personal and home environment variables examined. Click here for file

Additional file 2 **Distribution**^**a **^**of the current survey population**^**b **^**according to parental educational level and ethnic background.** Click here for file
