# Supplementary material for: Differences in beliefs and home environments regarding energy balance behaviors according to parental education and ethnicity among schoolchildren in Europe: the ENERGY cross sectional study
Source: BMC Public Health. 2014 Jun 17;14:610. doi: 10.1186/1471-2458-14-610 (PMC4067068; doi:10.1186/1471-2458-14-610)
Supplement: Additional file 1 — Overview of personal and home environment variables examined. [file 1471-2458-14-610-S1.docx]

Additional file 1. Overview of personal and home environment variables examined

| **CORRELATES** | **QUESTION FROM CHILD QUESTIONNAIRE** | **RESPONSE CATEGORIES**  **CODED AS 0**  **Positive/ favorable** | **RESPONSE CATEGORIES**  **CODED AS 1**  **Negative/ unfavorable** |
| --- | --- | --- | --- |
| **Soft drink** | | | |
| Unfavorable attitude | I think that drinking fizzy drinks or fruit squash is… | Very bad/ bad/  not bad, not good | Very good/ good |
| Incorrect health beliefs | I think that drinking fizzy drinks or fruit squash will make me fat | Fully agree/ somewhat agree/  do not disagree, do not agree | Fully disagree/  somewhat disagree |
| High preference/ liking | I like the taste of fizzy drinks or fruit squash | Fully disagree/ somewhat disagree/ do not disagree, do not agree | Fully agree/  somewhat agree |
| Unfavorable parental  subjective norm | If I drink fizzy drinks or fruit squash my parents/ caregivers  think this is… | Very bad/ bad/  not bad, not good | Very good/ good |
| Low parent modeling | How often do your parents/ caregivers drink fizzy drinks or fruit squash | Never/ not often/ sometimes | Always/ often |
| Parental practices |  |  |  |
| *Rules* | Do your parents/ caregivers have rules about how many fizzy drinks  or fruit squash you are allowed to drink | Yes | No |
| *High allowance* | I am allowed to take fizzy drinks or fruit squash, whenever I want | Never/ not often/ sometimes | Always/ often |
| *Bought on request* | If you ask your parents/ caregivers to buy a certain brand  of fizzy drink or fruit squash, will they do it | Never/ not often/ sometimes | Always/ often |
| *High accessibility* | If I ask my parents/ caregivers for a fizzy drink or fruit squash, I get one | Never/ not often/ sometimes | Always/ often |
| High home availability | Are there usually fizzy drinks or fruit squash at your home | Never/ not often/ sometimes | Always/ often |

| **CORRELATES** | **QUESTION FROM CHILD QUESTIONNAIRE** | **RESPONSE CATEGORIES**  **CODED AS 0**  **Positive/ favorable** | **RESPONSE CATEGORIES**  **CODED AS 1**  **Negative/ unfavorable** |
| --- | --- | --- | --- |
| **Breakfast** | | | |
| Unfavorable attitude | I think that eating breakfast is… | Very good/ good/  not bad, not good | Very bad/ bad |
| Health beliefs |  |  |  |
| *Incorrect   (eating breakfast)* | I think NOT eating breakfast will make me fat | Fully agree/ somewhat agree/  do not disagree, do not agree | Fully disagree/  somewhat disagree |
| *Incorrect  (not eating breakfast)* | I think that eating breakfast will make me fat | Fully disagree/ somewhat disagree/ do not disagree, do not agree | Fully agree/  somewhat agree |
| Low preferences/ liking | I like eating breakfast | Fully agree/ somewhat agree/  do not disagree, do not agree | Fully disagree/  somewhat disagree |
| Unfavorable parental  subjective norm | If I eat breakfast my parents/ caregivers think this is… | Very good/ good/  not bad, not good | Very bad/ bad |
| Low parent modeling | How often do your parents/ caregivers eat breakfast | Always/ often/ sometimes | Never/ not often |
| Low co-participation | How often do you eat breakfast with your parents/ caregivers | Every day/ 5-6 days a week/  2-4 days per week | Never/ less than once a week/  once a week |
| Low levels of active  encouragement/  parental support | My parents/ caregivers encourage me to have breakfast | Fully agree/ somewhat agree/  do not disagree, do not agree | Fully disagree/  somewhat disagree |
| Parental practices |  |  |  |
| *Rules* | Do your parents/ caregivers have rules about whether  you should eat breakfast | Yes | No |
| *Bought on request* | If you ask your parents/ caregivers to buy a certain brand of  food or drink for breakfast, will he/ she do it | Never/ not often/ sometimes | Always/ often |
| Low home availability | Are there usually breakfast products at your home | Always/ often/ sometimes | Never/ not often |

| **CORRELATES** | **QUESTION FROM CHILD QUESTIONNAIRE** | **RESPONSE CATEGORIES**  **CODED AS 0**  **Positive/ favorable** | **RESPONSE CATEGORIES**  **CODED AS 1**  **Negative/ unfavorable** |
| --- | --- | --- | --- |
| **Physical activity/ sports** | | | |
| Unfavorable attitude | I think that physical activity/ sports is… | Very good/ good/  not bad, not good | Very bad/ bad |
| Incorrect health beliefs | I think NOT doing physical activities/ sports will make me fat | Fully agree/ somewhat agree/  do not disagree, do not agree | Fully disagree/  somewhat disagree |
| Low preferences/ liking | I like doing physical activity/ sports | Fully agree/ somewhat agree/  do not disagree, do not agree | Fully disagree/  somewhat disagree |
| Unfavorable parental  subjective norm | If I do physical activity/ sports my parents/ caregivers think this is… | Very good/ good/  not bad, not good | Very bad/ bad |
| Low parent modeling | How often do your parents/ caregivers do physical activity/ sports | Always/ often/ sometimes | Never/ not often |
| Low co-participation | How often do you take part in physical activity/ sports with  your parents/ caregivers | Every day/ 5-6 days per week/  2-4 days per week | Never/ less than once a week/  once a week |
| Low levels of active  encouragement/  parental support | My parents/ caregivers encourage me to do physical activity/ sports | Fully agree/ somewhat agree/  do not disagree, do not agree | Fully disagree/  somewhat disagree |
| Parental practices |  |  |  |
| *Rules* | Do your parents/ caregivers have rules about whether  you should be physically active/ do sports | Yes | No |
| *Low general allowance* | Do your parents/ caregivers allow you to take part in  physical activity/ do sports | Yes | No |
| *Low specific allowance* | If you indicate that you like a certain physical activity/ sports  will your parents/ caregivers allow you to do it | Always/ often/ sometimes | Never/ not often |
| Low home availability | Do you have the following things at home that you can use for  physical activities/ sports: 1) bike, 2) tennis/ badminton racket, 3) ball,  4) sport shoes, 5) skipping rope, 6) skates, 7) skis, 8) skate board | Equal to or over 6 items | Less than 6 items |

| **CORRELATES** | **QUESTION FROM CHILD QUESTIONNAIRE** | **RESPONSE CATEGORIES**  **CODED AS 0**  **Positive/ favorable** | **RESPONSE CATEGORIES**  **CODED AS 1**  **Negative/ unfavorable** |
| --- | --- | --- | --- |
| **TV viewing** | | | |
| Unfavorable attitude | I think that watching TV is… | Very bad/ somewhat bad/  not good, not bad | Very good/ good |
| Incorrect health beliefs | I think watching too much TV will make me fat | Fully agree/ somewhat agree/  do not disagree, do not agree | Fully disagree/  somewhat disagree |
| High preferences/ liking | I like watching TV | Fully disagree/ somewhat disagree/ do not disagree, do not agree | Fully agree/ somewhat agree |
| Unfavorable parental  subjective norm | If I watch TV my parents/caregivers think this is… | Very bad/ somewhat bad/  not good, not bad | Very good/ good |
| High parent modeling | How often do your parents/ caregivers watch TV | Never/ not often/ sometimes | Always/ often |
| High co-participation | How often do you watch TV with your parents/ caregivers | Never/ less than once a week/  once a week | Every day, more than once/  every day, once a day/ 5-6 days per week/ 2-4 days per week |
| Parental practices |  |  |  |
| *Rules* | Do your parents/ caregivers have rules about how many hours per day  you are allowed to watch TV | Yes | No |
| *High general  allowance* | My parents/ caregivers allow me to watch TV whenever I want | Fully disagree/ somewhat disagree/ do not disagree, do not agree | Fully agree/ somewhat agree |
| *High specific  allowance* | If I ask my parents/ caregivers to watch TV, I can do so | Never/ not often/ sometimes | Always/ often |
| High home availability | Do you have a TV in your bedroom | No | Yes |
