# Supplementary material for: Differences in beliefs and home environments regarding energy balance behaviors according to parental education and ethnicity among schoolchildren in Europe: the ENERGY cross sectional study
Source: BMC Public Health. 2014 Jun 17;14:610. doi: 10.1186/1471-2458-14-610 (PMC4067068; doi:10.1186/1471-2458-14-610)
Supplement: Additional file 2 — Distribution a of the current survey population b according to parental educational level and ethnic background. [file 1471-2458-14-610-S2.docx]

**Additional file 2**. Distribution^a^ of the current survey population^b^ according to parental educational level and ethnic background

| **Country** | **Parental education**^c^ | | **Parental ethnic background**  **(language spoken at home)**^d^ | | **Parental ethnic background**  **(country of birth of biological parents)**^e^ | |
| --- | --- | --- | --- | --- | --- | --- |
|  | **% Low** | **% High** | **% Non-native** | **%Native** | **% Non-native** | **% Native** |
| Belgium | 14.6 | 85.4 | 6.8 | 92.6 | 8.2 | 91.6 |
| Greece | 46.8 | 53.2 | 8.2 | 91.7 | 29.5 | 67.7 |
| Hungary | 38.8 | 61.2 | 2.2 | 97.7 | 4.7 | 95.2 |
| the Netherlands | 20.7 | 79.3 | 4.5 | 95.5 | 15.2 | 84.0 |
| Norway | 23.3 | 76.7 | 3.7 | 95.9 | 15.7 | 84.3 |
| Slovenia | 41.8 | 58.2 | 7.4 | 92.2 | 19.9 | 79.9 |
| Spain | 18.5 | 81.5 | 2.5 | 97.2 | 10.2 | 89.5 |
| Switzerland | 56.2 | 43.8 | 20.4 | 79.2 | 34.1 | 64.9 |

^a^ Due to rounding errors and missing data, displayed percentages may not add up to 100

^b^ See also Brug et al 2012 [1] and Brug et al 2012 [5] for numbers on parental education and ethnic background in the ENERGY study;

^c^ Parental education was dichotomized into low (both parents/ caregivers with fewer than 14 years of education) and high (at least one parent/ caregiver with 14 or more years of education);

^d^ Children who reported to primarily speak the official language of the country of administration were classified as ‘native’, all other children were classified as ‘non-native’;

^e^ Children were classified as ‘native’ if both parents were born in the country of administration, all other children were classified as ‘non-native’.
